# High Load With Lower Repetitions vs. Low Load With Higher Repetitions: The Impact on Asymmetry in Weight Distribution During Deadlifting

**DOI:** 10.3389/fspor.2020.560288

**Published:** 2020-09-22

**Authors:** Mitchel C. Whittal, Derek P. Zwambag, Luke W. Vanderheyden, Greg L. McKie, Tom J. Hazell, Diane E. Gregory

**Affiliations:** ^1^Kinesiology and Physical Education, Wilfrid Laurier University, Waterloo, ON, Canada; ^2^Health Sciences, Wilfrid Laurier University, Waterloo, ON, Canada

**Keywords:** biomechanics, injury & prevention, kinetics, resistance, training

## Abstract

This study investigated weight distribution between the lower limbs using a symmetry index (SI) score of the vertical ground reaction forces (GRF) and measures of postural stability in high load/low repetition (termed “heavy”) and low load/high repetition (termed “light”) deadlifting. Ten participants performed two deadlift protocols with equal cumulative external load. These protocols were designed to represent standard high load/low repetition and low load/high repetition workouts; order was random and separated by 7 days. An effect of lifting condition (*p* = 0.023) and set number (*p* = 0.011) was observed such that lifts in the heavy condition were less symmetrical than those in the light condition and lifts became more symmetrical as set number increased. There was no effect of lift number on symmetry of force production (*p* = 0.127). Additional analysis revealed that center of pressure (COP) path length was greater during heavy lifts (*p* = 0.002) however COP range was unaffected suggesting controlled point of force application within the same boundaries regardless of lifting condition. As asymmetries have been previously associated with increased injury risk, greater training emphasis on the symmetrical performance of sub-maximal deadlifts should be considered to try to minimize the development of asymmetries.

## Introduction

A conventional deadlift is a compound movement combining concentric, isometric, and eccentric contractions to lift an anteriorly displaced weight from the ground to upright stance. The deadlift is an essential compound movement utilized for resistance training, rehabilitation, and powerlifting (Escamilla et al., 2000). The deadlift activates and builds musculature of the posterior chain and core (Hamlyn et al., 2007)—including but not limited to the transverse abdominis, rectus abdominis, erector spinae, latissimus dorsi, trapezius, gluteus maximus, and hamstrings—contributing to athletic performance, strength, and rehabilitation for low-intensity low back pain (Berglund et al., 2015; Thompson et al., 2015).

Functional asymmetries occur naturally when performing symmetrical tasks. Individuals naturally develop with a limb that is stronger and bears more load than the other limb, which is usually more coordinated (Ross et al., 2004). Bilateral asymmetries in arm muscle activation during the deadlift have been observed in lifters using a mixed or alternating grip (Beggs et al., 2014). Bilateral asymmetries have also been shown to persist during the performance of the barbell back squat (Sato and Heise, 2012), leading to increased tilting and rotation of the barbell. Analysis of symmetrical and staggered lifting stances found that one limb was loaded more than the other, and people seem to preferentially load their non-dominant limb (Brown and Reiser, 2012). For the most part, previous research has shown that these asymmetries do not negatively impact performance of the task in question (Lake et al., 2010, 2011; Dos'Santos et al., 2018), though not for all tasks and populations (Bishop et al., 2018).

Despite the unlikelihood that asymmetries will affect performance, asymmetrical production of force during dynamic resistance exercises, especially those that involve large external loads, may unintentionally alter lifting technique and cause excessive unilateral loading. Functional asymmetries of the lower limbs are believed to be related to an increased risk of injury (Hewett et al., 2001; Reiser et al., 2006) as well as a known risk factor for low back injuries (Choi et al., 2009). Despite this, the presence of low back pain (Zahraee et al., 2014), fatigue (Hodges et al., 2011), and previous unilateral injury (Reiser et al., 2006) do not appear to impact the symmetry of force production in the lower limbs during various tasks (e.g. standing, squat lifting). While the presence of low back (Zahraee et al., 2014) and unilateral injury (Reiser et al., 2006) do not further exacerbate functional asymmetries, they may contribute to the development of musculoskeletal disorders, especially when subjecting the body to large forces when lifting. The lifting of an external load—like a barbell during a conventional deadlift—greatly increases demands on muscular tissues; which may exaggerate the magnitude of functional asymmetries and potentially increase injury risk. Despite this, strength is built through resistance training, and lower body strength is known to protect against injury (Askling et al., 2003; Trudelle-Jackson and Morrow, 2011).

Recreational lifting is often performed either with a heavy weight and low repetitions, or light weight with high repetitions. Similar hypertrophic gains can be achieved with low or high load training, but maximal strength is better obtained through using higher loads (Schoenfeld et al., 2017), and greater training volumes attained with lower loads Schoenfeld et al. (2016). However, it is unknown how these different lifting profiles alter the symmetry of force production and whole-body stability. The purpose of this research was to investigate the differences in weight distribution between the lower limbs using a symmetry index score of the vertical ground reaction forces (GRF) and measures of postural stability in high weight/low repetition and light weight/high repetition deadlifting. It was hypothesized that individuals would be less symmetrical and have greater COP ranges during the heavy condition compared to the light condition.

## Materials and Methods

### Subjects

Ten participants (9 male and 1 female) with experience performing the deadlift with no history of back pain (previous 6 months) and no musculoskeletal disorders were recruited. Due to failure to conform to lifting instructions (failure to pause/reset between repetitions resulting in a bouncing of the barbell), one participant was removed from analyses. Examination of raw data and residuals indicated that the single female participant was not a statistical outlier.

Participants had a mean age (mean ± SD) of 21.7 ± 2.9 years, mass of 79 ± 9.4 kg and height of 180.7 ± 8.9 cm. All participants were university students recruited through poster advertisements. Participants had an average estimated 1-repetition maximum (1RM) of 149.58 ± 37.26 kg. Each participant reviewed and signed the consent form approved by the University Research Ethics Board.

### Instrumentation

**Figure 1:**
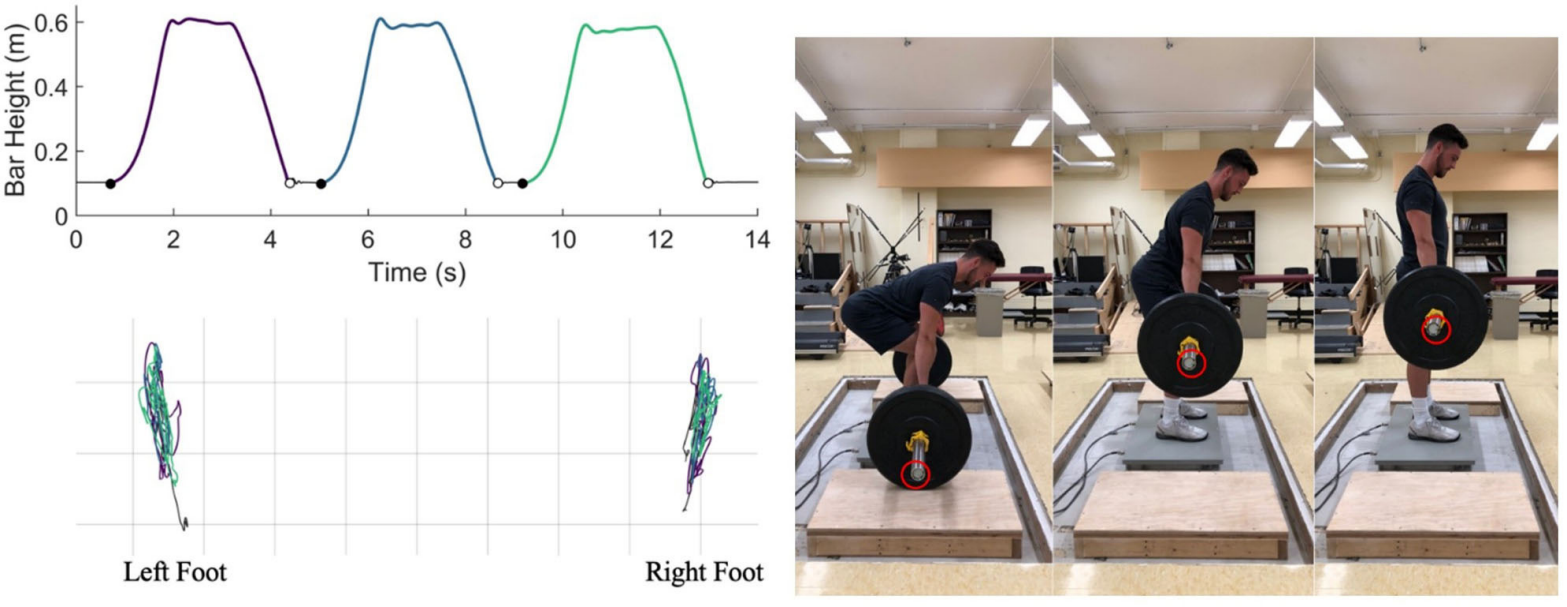
Overview of the lifting protocol: Top left: Kinematic detection of start (filled circle) and end (open circle) of each lift during a 3-repetition deadlift set. Bottom left: Trajectory of center of pressure under left and right feet throughout the set. Grid squares are 5 cm^2^. Different colors represent three different lifts in both plots. Right: Completion of the concentric portion of the deadlift depicted with a neutral head and spine position until full hip and knee extension are reached. The location of the kinematic marker is indicated by the red circle.

Two force plates (model no. OR6-7-2000; AMTI, Watertown, MA, USA) were used to collect GRF and moments beneath each foot (sample rate of 1,000 Hz) during the deadlifts to calculate COP variables. From a sagittal view, lift detection was determined using the vertical position and velocity of the barbell acquired from an active infrared kinematic marker via the Optotrak camera system (Optotrak, Northern Digital Inc, Waterloo, ON, Canada) fixed to the end of the barbell (Figure 1). Lift initiation and cessation was determined using the directions of the vertical velocity of the bar. The height of the barbell was differentiated with respect to time to get vertical velocity. To exclude small movements of the bar before, after, or between lifts, lift initiation was detected by identifying instances of bar height reaching 25% of its maximum and then searching prior to each instance for the first frame where vertical velocity was positive. Similarly, lift cessation was determined by identifying when the barbell was 75% of the way down from max height and then searching forward in time for the last frame where vertical velocity was negative. Position data were synchronized with force plate data via the use of an external trigger and collected in AMTI's NetForce software. Foot location was marked to enable consistent placement during each set within each testing condition. Force plate data were filtered with a low-pass 4th order dual pass Butterworth filter with an effective cut-off of 10 Hz.

### Lifting Procedures

Prior to the first collection day, participants completed a 5-repetition maximum test with a weight they could lift five times without technique breakdown. Technique breakdown was characterized by excessive lumbar or thoracic rounding, excessive cervical extension, and/or significant rising of the hips prior to barbell movement. The achieved load was used to determine the estimated 1RM of each participant using the following from the Epley equation (Epley, 1985):

$$\begin{array}{l} 1RM\;=\;Weight\;\times \;\left[1+\left(0.033\times number\;of\;repetions\right)\right] \end{array}$$

Prior to either lifting condition, participants completed a warm-up which consisted of 10 × 20 kg followed by 5 × 61 kg. Following their warm-up, the lifting protocol was performed; lifting conditions are described in Table 1. The light condition involved 6 × 50% 1RM followed by two working sets of 10 × 60% 1RM. The heavy condition involved 2 × 6 50% 1RM, 2 × 70% 1RM, and then three working sets of 3 × 85% 1RM. The cumulative external load was 1,500% 1RM for the light condition and 1,505% 1RM for the heavy condition. Each warm-up set had a rest time of 2 min and each working set had a rest time of 5 min.

**Table 1:** Lifting protocol for each test condition ensuring similar cumulative external load between conditions.

**Warm Up**	
**1 set of 10 repetitions × 20 kg**	
**1 set of 5 repetitions × 61 kg**	
**Light**	**Heavy**
1 set of 6 repetitions × 50% 1RM	2 sets of 6 repetitions × 50% 1RM 1 set of 2 repetitions × 70% 1RM
2 sets of 10 repetitions × 60% 1RM ***Cumulative external load = 1,500% of 1RM***	3 sets of 3 repetitions × 85% 1RM ***Cumulative external load = 1,505% of 1RM***

Participants were given a brief overview of the lifting condition (light or heavy; randomized) that they would perform that day. Participants were instructed to perform deadlifts while maintaining a neutral head and spine position during each repetition until hip and knee extension were reached. Each participant was told to lower the barbell to a complete stop between each repetition to isolate each lift (Figure 1). Verbal encouragement was provided in addition to participants being told the remaining number of lifts that they were to perform each set. Participants were not provided with any feedback signals regarding symmetry or force production of their deadlift performance. The use of weightlifting chalk was available and recommended (if deemed necessary) by a researcher monitoring the performance of lifts. All participants were asked to use a mixed grip—grasping the barbell with one supinated and one pronated hand—for the heaviest sets to prevent the barbell from rolling or slipping in-hand. One participant utilized a lifting belt with no resulting differences in symmetry scores. Testing conditions were separated by 1 week with both sessions completed at the same time of day. Participants were required to refrain from extraneous physical activity for 24 h prior to each data collection to eliminate possible fatigue or soreness effects. Participants indicated their level of exertion (rating of perceived exertion on a 6–20 Borg scale) after completing each set of 60 and 85% lifts (Williams, 2017).

### Data Analysis

A symmetry index score of the vertical GRF (Sato and Heise, 2012) was used to determine the symmetry of force production between the lower limbs, where:

$$\begin{array}{l} SI=\frac{\left|GRF_{L\;}-GRF_{R}\right|}{GRF_{L\;}+GRF_{R}}\times 100 \end{array}$$

**Figure 2:**
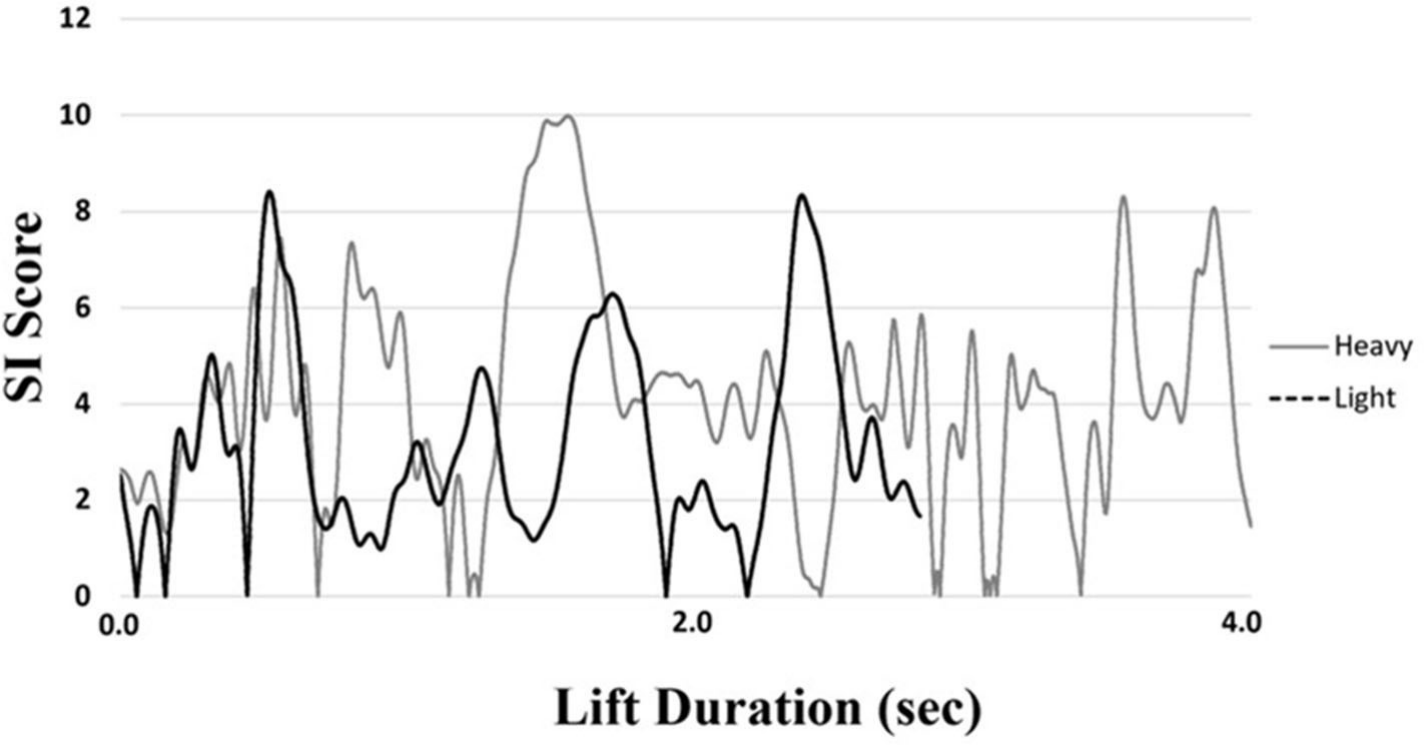
Representative depiction of the progression of SI score throughout the performance of a lift in the heavy and light conditions of a single participant. Average SI score was calculated for each separate lift and utilized for analysis. Note that the lift in the heavy trial took more time to complete than the lift in the light condition.

A symmetry score of zero would indicate perfect symmetry of weight distribution under the lower limbs; scores greater than zero indicate some degree of asymmetry with higher SI scores indicating greater asymmetry. SI scores were calculated for the 60% 1RM sets in the light condition and the 85% 1RM sets in the heavy condition. Further, SI scores were determined from the total motion (concentric and eccentric) of the lift as the isolated concentric and eccentric SI scores were found to be strongly correlated with the total lift SI scores in the current dataset (*r* = 0.85 and *r* = 0.90, respectively). An average SI score was calculated for each repetition of each set and condition (Figure 2). COP range data were used to describe movement of the COP within and across lifts. COP path length was calculated as the Euclidean distance of two-dimensional COP coordinates, for a representation of total distance traveled that is independent of anterior-posterior and medial-lateral distinction. Average COP velocity was calculated by dividing the total COP path length by the time to complete each repetition.

### Statistical Analyses

A linear mixed effects ANOVA model was used to determine the effects of lifting condition, set number, lift number, and participant on SI scores, lift duration, COP range, COP velocity, and COP path length. Lifting condition, set number, and lift number were all fixed effects. Participant was included as a random factor to account for repeated measures. Accepted level of significance was set at α = 0.05. All results are presented as average [standard error of the mean (SEM)], with effect sizes (ES) for between-condition comparisons. Pearson's correlation was conducted to identify possible relationships between outcome variables and remove redundancy of reported results. Correlations > *r* = 0.70 were considered strong. Effect size thresholds of 0.1, 0.3, 0.5, 0.7, and 0.9 were considered small, moderate, large, very large, and extremely large (Cohen, 1988; Hopkins et al., 2009).

## Results

**Figure 3:**
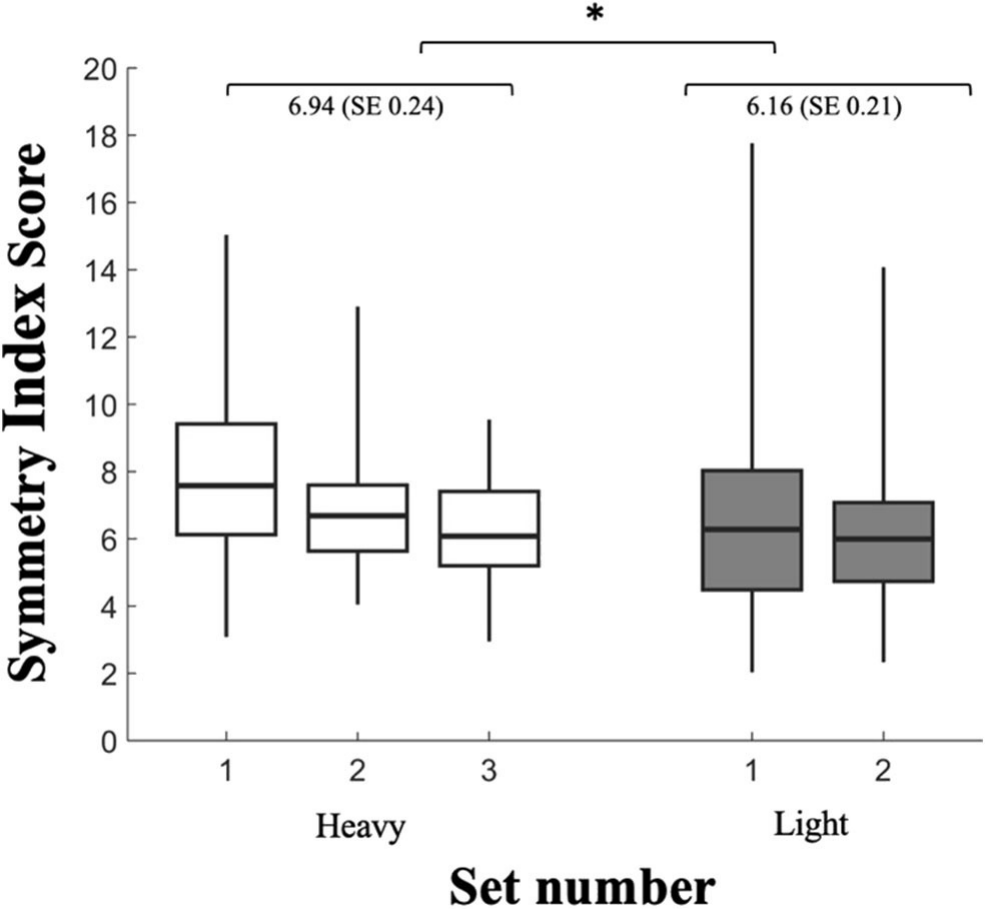
Symmetry index scores for each set of heavy (white) and light (gray) deadlifts. Box extends from 25th to 75th percentiles and are bisected by the median. Whiskers extend to maximum and minimum symmetry index scores. Mean and SE for light and heavy conditions listed. Significance denoted * as *p* < 0.05. Note that while between participant variability was quite high, given the repeated measures design of the study, differences in the SI score between conditions were still found to be significant (*p* = 0.023) indicating a similar response across the nine participants.

Analysis of variance revealed an effect of set number (*p* = 0.011) and condition (*p* = 0.023; ES 0.52), such that SI scores decreased by an average of 1.66 each set, with average SI scores showing 11.23% greater symmetry in the light condition than heavy (Table 2). Interestingly, as shown in Figure 3, between participant variability was quite high, however, differences in the SI score between conditions were still found to be significant (*p* = 0.023) indicating a similar response across the nine participants. There was no effect of lift number on SI scores (*p* = 0.127) and no significant interactions between condition, set number, or lift number (*p* > 0.05).

**Table 2:** Summary of outcome measures and their main effects.

**Measure**	**Mean (SEM) across condition**	**Dependent Variable**	**Significance (*p*)**
SI Score	***Heavy: 6.94 (0.24)*** ***Light: 6.16 (0.21)***	Condition	0.023^*^
		Set number	0.011^*^
		Lift number	0.127
AP COP Range(mm)	Heavy: 99.67 (3.47) Light: 93.04 (2.27)	Condition	0.289
		Set number	0.780
		Lift number	0.832
ML COP Range(mm)	Heavy: 22.99 (1.37) Light: 22.21 (0.91)	Condition	0.977
		Set number	0.840
		Lift number	0.980
COP Path Length(mm)	***Heavy: 535.45 (16.0)*** ***Light: 432.41 (7.2)***	Condition	0.002^*^
		Set number	0.196
		Lift number	0.738
Average COP Velocity (mm/s)	Heavy: 177 (5.55) Light: 174.83 (2.99)	Condition	0.061
		Set number	0.121
		Lift number	0.047^*^
Lift Duration(s)	Heavy: 3.09 (0.07) Light: 2.54 (0.04)	Condition	0.051
		Set number	0.99
		Lift number	0.034^*^

* *Denotes statistical significance at the α = 0.05 level. Mean values shown in bold were found to be significantly different from each other (p < 0.05)*.

COP ranges under right and left feet were highly correlated in anterior-posterior (*r* = 0.81) and medial-lateral (*r* = 0.73) directions; therefore, data from each foot were pooled for further analysis. In the anterior-posterior direction, there was no effect of set number (*p* = 0.780), lift number (*p* = 0.832), or condition (*p* = 0.289; ES 0.22) as AP range was 6.65% smaller in the light condition (Table 2). Additionally, there was no effect of set number (*p* = 0.840), lift number (*p* = 0.980), or condition (*p* = 0.977; ES 0.07) on pooled measures of medial-lateral COP range with only a 3.39% change in ML range between heavy and light conditions, respectively.

A significantly greater COP path length (*p* = 0.002; ES 0.84) was observed in the heavy condition resulting from a 19.24% reduction in path length in the light condition (Table 2). There was no effect of lift number (*p* = 0.738) or set number (*p* = 0.196) on COP path length.

**Figure 4:**
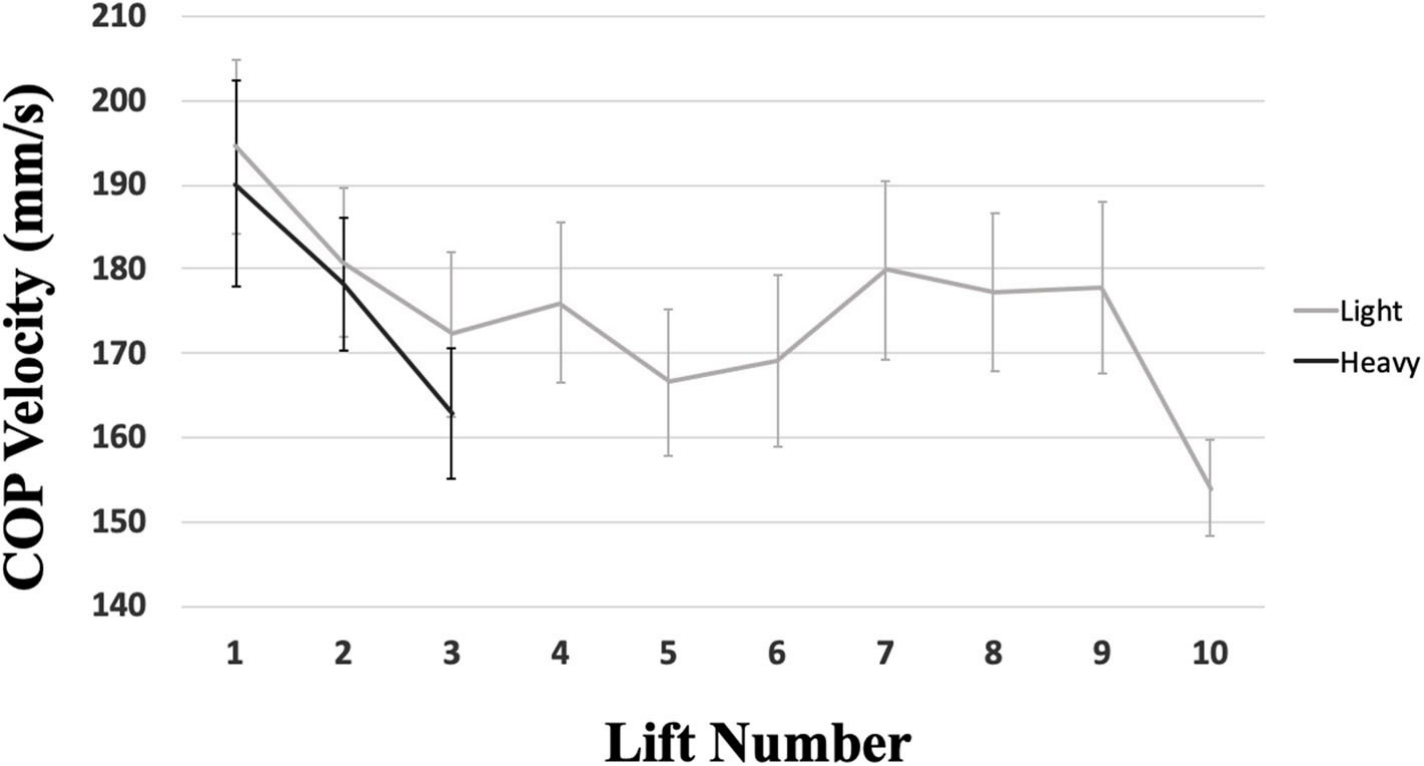
COP velocity for each lift, separated by condition. Error bars indicate standard error. COP velocity decreased as lift number increased (*p* = 0.047) and a trend toward an effect of lifting condition (*p* = 0.061).

**Average** COP velocity decreased as lift number increased (*p* = 0.047; Figure 4). There was insufficient evidence to support a main effect of lifting condition (*p* = 0.061; ES 0.05) or set number (*p* = 0.121) on COP velocity; the average velocity was only 1.23% slower in the light condition (Table 2).

There was an effect of lift number on lift duration (*p* = 0.034) such that lifts took an average of 0.1633 s longer for each increase in lift number. Lifts in the light condition took 17.80% less time to complete (Table 2); however, this only reached near significance (*p* = 0.051; ES 0.94).

There were no observed differences in perceived exertion between conditions (*p* = 0.929; ES 0.03), with means of 13.24 (SEM 0.31) and 13.19 (SEM 0.43) in the heavy and light conditions, respectively.

## Discussion

Analysis of SI scores identified a main effect of set number and lifting condition (light vs. heavy), supporting the hypothesis that participants would be less symmetrical when performing heavy deadlifts for fewer repetitions compared to light deadlifting for more repetitions. Data did not support the hypothesis that lifts in the heavy condition would result in greater COP ranges as anterior-posterior and medial-lateral measures of COP range were not impacted by condition, set number, or lift number. However, heavy lifts did result in larger COP path lengths. Increased COP path length with no change in COP range indicated that participants were controlling their point of force application within the same boundaries regardless of lifting condition. Despite similar COP ranges between conditions, greater COP velocities were found during lifts performed in the heavy condition, supporting the original hypothesis.

The main finding of this analysis was that participants exerted force less symmetrically when asked to perform three repetitions with 85% of 1RM compared to performing 10 repetitions with 60% of their 1RM. Decreased force symmetry with an increase in intensity could possibly be explained as the exaggeration of existing muscular asymmetries by resorting to exerting as much force as possible with each limb (Ross et al., 2004). Alternatively, it is possible that lifters were not able to focus as much on equal force distribution and proper technique when performing the higher intensity 85% lifts. Both the 60 and 85% intensities displayed a decrease in the SI score as set and lift number increased, indicating that participants were becoming more symmetrical in their force production within each set and throughout the workout. Although there was no effect of lift number on SI scores (*p* = 0.127), participants COP velocity was the most variable during the first repetition of each set, indicating that there could be greater challenges to postural control during the first repetition of a lifting set.

Interestingly, there did not appear to be a negative effect of performing subsequent sets and repetitions on measures of symmetry. In fact, regression analysis determined symmetry index scores improved as set and lift number increased. Improved symmetry throughout the progression of the workout suggests that there may have been a practice or learning effect to performing deadlifts at a specified load such that each participant became familiarized with the weight and as a result, symmetry improved. Similarly, analysis of force asymmetries during the squat found that asymmetries decreased within each set and that healthy individuals load their limbs more symmetrically as the workout progressed (Hodges et al., 2011). Practically, this result suggests that individuals should perform adequate warm-ups focusing on proper technique and symmetry prior to engaging in heavier lifts as the progression of a workout did not appear to negatively alter limb loading.

The results excluded from the individual who did not follow lifting instructions also revealed noteworthy asymmetry while lifting. This participant did not rest between repetitions during the light condition, but rather “bounced” the weight on the floor between repetitions. The result was an SI score of 11.06 in the light condition, compared to the average 6.16, and was almost six times greater than the standard deviation of the remaining nine participants in that condition. The asymmetrical loading profile (see Supplementary Material) of this individual, which was more asymmetrical than the other participants, suggests that lifting in an uncontrolled fashion may also decrease whole-body stability and thereby potentially increase risk of injury. This statement should be interpreted with caution as this finding was only present with a single individual and may not represent the findings of a greater sample.

The current research must be taken with a number of considerations. Participants wore their own choice of footwear; potentially impacting COP deviations between participants due to differences in sole stiffness and stability of force application. The selection of 60 and 85% of 1RM likely diminished differences in SI scores between conditions, however; these loads were selected to represent realistic conditions that lifters utilize in resistance training, increasing external validity. As aberrant lifting technique is a possible driver for asymmetry, the measurement of altered joint angles and barbell tilt could provide greater context to the extent and cause of asymmetry present during the deadlift. Additionally, analysis of advanced lifters would be valuable to determine if weight distribution asymmetries persist in a sample with a high degree of technical proficiency, as this population may be more accustomed to lifting loads closer to their 1RM. In addition to the examination of advanced lifters and added kinematic analyses, future research should examine the effect of set number and load variation on symmetry scores. A comparison of static load and varied load across a fixed number of lifting sets would elucidate the effects of set number vs. the number of sets with the same load, or load familiarization.

Asymmetrical performance of deadlifts could reinforce muscular imbalances and functional asymmetries; potentially leading to imbalanced hypertrophy and strength. It has been documented that lower limb strength imbalances exist within athletic populations (Newton et al., 2006; Blache and Monteil, 2012; Atkins et al., 2016; Fuller et al., 2017), and that imbalances can and should be corrected through training to minimize injury risk (Bazyler et al., 2014; Bailey et al., 2015a,b). If uncorrected, asymmetries can have detrimental consequences to the athletic population. Functional asymmetry identification prior to in-season competition was predictive of injury in both American and Australian football athletes (Kiesel et al., 2007, 2014; Chalmers et al., 2017). Contralateral differences in knee flexor strength increased risk of injury, and discrepancies in hip flexor strength have been identified as a risk factor for low back pain (Knapik et al., 1991; Nadler et al., 2001). Despite this, there is likely no specific threshold below which asymmetry is tolerable as the risk of injury depends greatly upon the performed task, as well as the characteristics of each individual executing the task. Future research should consider further examining the relationship between lower limb symmetry and injury prevalence when lifting. Lastly, research should consider examining other measures of symmetry such as variance in the symmetry score and its impact on risk of injury.

If lifters seek to minimize the development of functional asymmetries, the current research indicates that deadlift training should be performed with loads closer to the 60% light condition to minimize force discrepancies in the lower limbs. A potential concern with recommending lower intensity lifting for individuals seeking athletic performance is reduced strength gains; to combat this, planned variations in load and volume can effectively be utilized to increase strength (Williams et al., 2017).

Regardless of the load used, each set should be treated with the same attention to technique to minimize the potential for injury and development of functional asymmetries. Further, the performance of more first lifts with a focus on symmetry should be prioritized if the goal is to lift as much weight as possible due to the lifters in this study displaying greater variability of biomechanical measures during initial lifts.

The current study observed increased lower limb weight distribution asymmetry during deadlifts with higher load and lower repetitions when compared to lower loads with higher repetitions. Given that asymmetries have been previously associated with increased injury risk, high load/low repetition deadlifting may pose a risk to athletes. Greater training emphasis on the symmetrical performance of sub-maximal deadlifts should also be considered to try to minimize the development of asymmetries.

## Data Availability Statement

The raw data supporting the conclusions of this article will be made available by the authors, without undue reservation.

## Ethics Statement

The studies involving human participants were reviewed and approved by Research Ethics Board, Wilfrid Laurier University. The patients/participants provided their written informed consent to participate in this study. Written informed consent was obtained from the individual(s) for the publication of any potentially identifiable images or data included in this article.

## Author Contributions

All authors listed have made a substantial, direct and intellectual contribution to the work, and approved it for publication.

## Conflict of Interest

The authors declare that the research was conducted in the absence of any commercial or financial relationships that could be construed as a potential conflict of interest.
